# DMDD, isolated from *Averrhoa carambola* L., ameliorates diabetic nephropathy by regulating endoplasmic reticulum stress-autophagy crosstalk

**DOI:** 10.1186/s13020-024-00993-z

**Published:** 2024-09-12

**Authors:** Jianmei Shi, Yuxiang Wang, Tao Liang, Xixi Wang, Jingxiao Xie, Renbin Huang, Xiaohui Xu, Xiaojie Wei

**Affiliations:** 1 Department of Physiology, College of Basic Medicine, Guangxi University of Chinese Medicine, Nanning, 530021 Guangxi China; 2 Guangxi Key Laboratory of Translational Medicine for Treating High-Incidence Infectious Diseases with Integrative Medicine, Nanning, 530021 Guangxi China; 3 Pharmaceutical College, Guangxi Medical University, Nanning, 530021 Guangxi China; 4 Key Laboratory of Research and Application of Stomatological Equipment (College of Stomatology, Hospital of Stomatology, Education Department of Guangxi Zhuang Autonomous Region, Guangxi Medical University), Nanning, 530021 Guangxi China; 5 Department of Pharmacy, Guangxi Medical University Cancer Hospital, Nanning, 530021 Guangxi China

**Keywords:** Diabetic nephropathy, 2-Dodecyl-6-Meth-Oxycyclohexa-2,5-Diene-1,4-Dione, Endoplasmic reticulum stress, Autophagy

## Abstract

**Background:**

Studies have shown that *Averrhoa carambola* L. possesses therapeutic potential for diabetes and related complications. However, the specific beneficial effects and molecular mechanisms of 2-dodecyl-6-meth-oxycyclohexa-2,5-diene-1,4-dione (DMDD) isolated from *Averrhoa carambola* L. on diabetic nephropathy (DN) require further investigation.

**Methods:**

80 C57BL/6 J male mice were subjected to a 1-week adaptive feeding, followed by a high-fat diet and intraperitoneal injection of 100 mg/kg streptozotocin (STZ) to construct an in vivo DN model. Additionally, human renal proximal tubular epithelial cells (HK-2) induced by high glucose (HG) were used as an in vitro DN model. The expression levels of epithelial-mesenchymal transition (EMT), endoplasmic reticulum stress (ERS), and autophagy-related proteins in renal tubular cells were detected by Western Blot, flow cytometry, immunofluorescence, and enzyme-linked immunosorbent assay (ELISA) staining. Transcriptome analysis revealed was conducted to elucidate the specific mechanism of by which DMDD mitigates DN by inhibiting ERS and autophagy. HK-2 cells were transfected with IRE1α overexpression lentivirus to reveal the role of IRE1α overexpression in HG-induced HK-2.

**Results:**

The experimental data showed that DMDD significantly reduced blood glucose levels and improved renal pathological alterations in DN mice. Additionally, DMDD inhibited the calcium (Ca^2+^) pathway, manifested by decreased autophagosome formation and downregulation of LC3II/I, Beclin-1, and ATG5 expression. Moreover, in HG-induced HK-2 cells, DMDD suppressed the overexpression of GRP78, CHOP, LC3II/I, Beclin1, and ATG5. Notably, IRE1α overexpression significantly increased autophagy incidence; however, DMDD treatment subsequently reduced the expression of LC3II/I, Beclin1, and ATG5.

**Conclusion:**

DMDD effectively inhibits excessive ERS and autophagy, thereby reducing renal cell apoptosis through the IRE1α pathway and Ca ^2+^ pathway.

## Introduction

Diabetic nephropathy (DN) is a serious microvascular complication of diabetes. With the rising prevalence of diabetes, the incidence of DN is also increasing. The classic symptom of DN is persistent proteinuria (PRO), followed by renal tubular cell damage, renal tubular interstitial damage, and eventually renal failure. Studies have shown that renal tubular damage may precede glomerular damage, and the degree of such damage is closely related to the prognosis of DN treatment [1]. In addition, renal tubular damage can lead to proteinuria and exacerbate the progression of DN [2, 3]. Although current clinical therapies can alleviate symptoms, the treatment of DN remains challenging. Therefore, it is very crucial to study the therapeutic mechanism of DN.

The endoplasmic reticulum (ER) is a key organelle responsible for protein synthesis, folding, and maintaining calcium homeostasis, all essential for maintaining intracellular homeostasis. Under continuous high glucose (HG) stimulation, the structure and function of the ER are impaired, resulting in excessive aggregation of defective proteins in the ER lumen, thereby causing endoplasmic reticulum stress (ERS). The body initiates self-protection signals to mitigate ERS-induced cellular damage and activates the unfolded protein response (UPR). IRE1α is one of the important channels in the UPR, activating its downstream proteins and regulating apoptotic gene expression [4, 5]. Autophagy is a rare adaptive metabolism in eukaryotic cells that regulates intracellular homeostasis [6]. There is a cross-talk effect between ERS and autophagy. Prolonged HG stimulation in renal tubular epithelial cells enhances ERS-autophagy crosstalk, which kills cells and accelerates the progression of DN [7–9]. As a result, alleviating excessive ERS and autophagy may be an effective strategy for treating DN.

2-dodecyl-6-methoxycyclohex-2,5-diene-1,4-dione (DMDD) is a flavonoid compound extracted and identified from carambola. It possesses hypoglycemic and anti-inflammatory properties, which can mitigate the progression of DN [10]. Previous study have shown that DMDD can alleviate renal injury and inflammation by regulating the TLR4/MyD88/NF-κB signaling pathway, thereby mitigating the pathogenesis of DN [11]. Another study from Li et al. indicated that DMDD effectively inhibits HG-induced epithelial-mesenchymal transition (EMT) in HK-2 cells by regulating the miR-21 / Smad7 axis and TGFβ1/Smad2/3 signaling pathways, thereby reducing renal fibrosis in DN [12]. Our molecular docking and protein multi-omics results indicate that DMDD is closely related to glucose metabolism-related proteins, the IRE1α pathway, and the Ca^2+^ pathway. Although DMDD can ameliorate DN and is potentially related to oxidative stress, renal fibrosis, and glucose metabolism, the molecular interaction and mechanisms between DMDD and ERS target genes remain unclear. Therefore, in this study, we used C57BL/6 mice injected with streptozotocin (STZ) to establish a DN model and HG-induced HK-2 cell models to explore the effects of DMDD on ERS-autophagy and its protective role against tubular epithelial cell damage.

## Materials and methods

### Plant material and extraction of DMDD

**Fig. 1:**
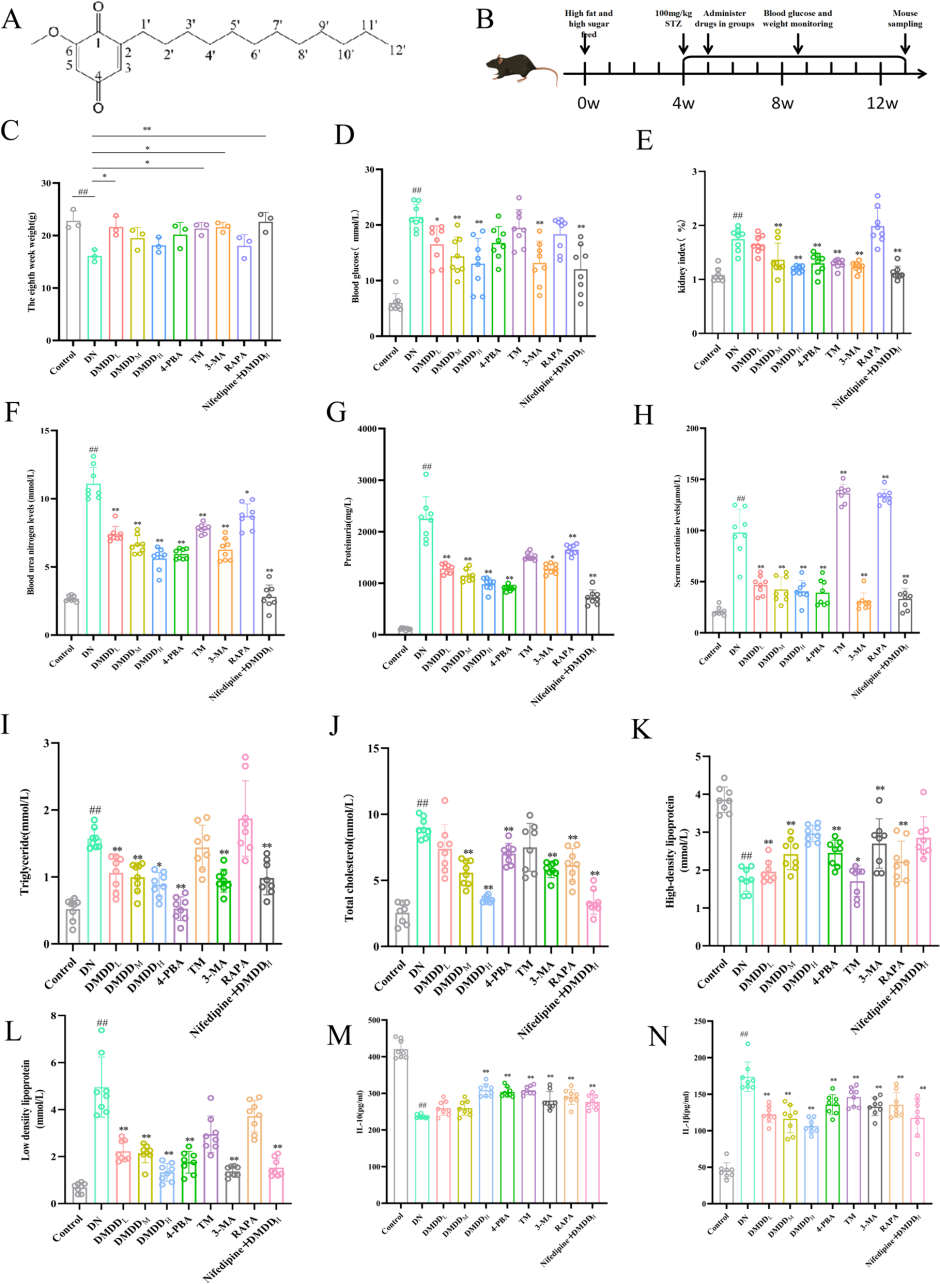
Effects of DMDD on renal function, blood lipids, and inflammatory factors in DN mice. **A** Chemical structure formula of DMDD; **B** Flowchart depicting the establishment of the in vivo DN model; **C** Body weight changes of mice in each group during the 8-week post-administration; **D** Blood glucose levels of mice in each group after 8 weeks of DMDD administration following successful establishment of the DN in vivo model; **E** Renal index changes in mice in each group after 8 weeks of DMDD administration following the successful modeling of the DN in vivo model; **F**–**H** Effects of DMDD administration on renal function in mice; **I**–**L** Effects of DMDD on blood lipids in mice after Nifedipine + DMDD administration; **M**–**N** Effects of DMDD administration on inflammatory factors IL-10 and IL1β levels. The Control group refers to the normal group, DN group to the diabetic nephropathy model group, DMDD_L_, DMDD_M_, and DMDD_H_ represent low-, medium-, and high-dose DMDD groups, respectively. Additionally, 4-PBA group denotes the endoplasmic reticulum stress inhibitor 4-PBA group, TM group denotes the endoplasmic reticulum stress agonist TM group, 3-MA group denotes the autophagy inhibitor 3-MA group, RAPA group denotes the autophagy agonist RAPA group, and Nifedipine + DMDD_H_ group denotes nifedipine group + DMDD high-dose group. Data are expressed as mean ± SD (n = 8). Statistical significance: compared to the normal group, ^#^*P* < 0.05, ^##^*P* < 0.01; compared to the model group, *P < 0.05, **P < 0.01

*Averrhoa carambola* L. was collected from Lingshan County, Guangxi Zhuang Autonomous Region, China. It was identified by Professor Mao Xianglai from the Guangxi Institute of Traditional Chinese Medicine. The voucher specimen (No. 20121016) was preserved in the herbarium of Guangxi Institute of Traditional Chinese Medicine (Guangxi, China). 10 kg of crude carambola root powder was mixed with a 60% ethanol-distilled water solution (medicinal materials: mixture = 1:8), and completely soaked for 1 h. Reflux extraction was first performed under normal pressure and then under reduced pressure to obtain a concentrated solution. The concentrated solution was extracted with ethyl acetate and cyclohexane, followed by elution with varying proportions of cyclohexane: ethyl acetate solution to obtain a yellow crystalline extract. The purity of DMDD, determined by high-performance liquid chromatography (HPLC), was ≥ 95%. The chemical structure of DMDD is depicted in Fig. 1A.

### Cell culture

Human renal proximal tubular epithelial cells, HK-2 cells, were purchased from Procell Life Science and Technology Co., Ltd. (Wuhan, China). HK-2 cells were cultured in a low-glucose medium (DMEM, Gibco, New York, USA) supplemented with 1% penicillin/streptomycin (Sorlabio, China) mixture and 10% fetal bovine serum. The cells were maintained in an incubator at 37 °C with 5% CO_2_. Upon reaching approximately 80% confluence, the HK-2 cells were digested with pancreatic enzymes, passaged, and subjected to related experiments.

The experimental groups were as follows: (1) control group: cells cultured in a low-glucose medium (glucose concentration of 5.56 mmol/L); (2) HG model group: cells cultured in a high glucose medium (glucose concentration of 60 mmol/L), treated for 48 h; (3) 4-PBA group: HG-treated cells added with 5 mmol/L 4-PBA; (4) TM group: HG-treated cells added with 5 μg/mL TM; (5) 3-MA group: HG-treated cells added with 5 mmol/L 3-MA; (6) RAPA group: HG-treated cells added with 10 μmol/L RAPA; and (7) DMDD group: HG-treated cells added with different doses of DMDD (8, 4, and 2 μmol/L).

### Animals and treatment

A total of 80 C57BL/6 mice (male, 17–20 g, SPF grade) were purchased from the Animal Experimental Center of Guangxi Medical University. The experimental animal production license number is SCXK (Gui) 2020–0003, and the experimental animal use license is SYXK Gui 2020–0004. All mice were raised in the Specific Pathogen Free (SPF) animal experimental center of Guangxi Medical University under the standard temperature (25 ± 2) °C with relative humidity (60 ± 10%) and a 12-h light/dark cycle. Mice were housed in separate cages with ad libitum access to food and water, and bedding and drinking water were replaced daily. After 1 week of adaptive feeding, 8 mice were randomly selected as the normal group and were fed with a normal diet, while the remaining mice were given a high-fat diet for 1 month (the high-fat diet was purchased from Beijing Bo Ai Gang Biotechnology Co., Ltd.). Following 3 days of fasting without water for 12 h, the tail vein blood was collected to detect the mice's fasting blood glucose (FBG). A successful model was confirmed if the FBG was ≥ 11.1 mmol/L.

Upon successful modeling, the diabetic mice were randomly divided into 6 groups: (1) 4-PBA (10 mg/kg/d), (2) TM (1 mg/kg/d), (3) 3-MA (15 mg/kg/48 h), (4) RAPA (2 mg/kg/48 h), (5) DMDD_H_ + Nifedipine (50 mg/kg/d + 20 mg/kg/d), and 6) DMDD high, medium, and low doses (50, 25, and 12.5 mg/kg/d, respectively). Each group had eight mice, and drugs were administered according to the respective group assignments. The normal group (set as control) and model groups were given equal volumes of distilled water, while 4-PBA, TM, 3-MA, and RAPA were administered intraperitoneally for 8 weeks. The remaining groups received continuous oral gavage for 8 weeks. During the treatment, in addition to the normal group receiving standard feeding, the remaining groups continued the high-fat diet. The mice's blood glucose and body weight were measured and recorded every 7 days. Mice in the normal group were fed with a normal diet, while mice in the other groups were administered corresponding drugs. The 3-MA group, RAPA group, 4-PBA group, and TM group were injected intraperitoneally. The DMDD low-dose, middle-dose, high-dose, and DMDD_H_ + Nifedipine groups were administered intragastric. The control and model groups were given equal volumes of distilled water. After 8 weeks of continuous administration, the urine samples of the mice were collected, and their FBG and body weight were measured. Blood samples were obtained through orbital bleeding, and the mice were sacrificed by cervical dislocation for kidney tissue collection. During the treatment, the normal group continued a standard diet, while the other groups maintained on a high-fat diet. The modeling process is depicted in Fig. 1B. These studies were approved by the Laboratory Animal Ethics Committee of Guangxi Medical University on May 10, 2021 (approval No. 202105088).

### Urine and blood analysis

The following kits from Nanjing Jiancheng Bioengineering Institute (Nanjing, China) were employed to detect serum creatinine (Scr), the blood urea nitrogen (BUN) determination kit (No. 20220829), the Scr determination kit (No. 20220922), the PRO quantitative test box (No. 20220922), the triglyceride (TG) determination kit (No. 20220830), the total cholesterol (T-CHO) determination kit (No. 20220830), the high-density lipoprotein cholesterol (HDL-C) kit (No. 20220830), and the low-density lipoprotein cholesterol (LDL-C) kit (No. 20220830).

### Histology and histopathology

Following dewaxing and dehydration, hematoxylin–eosin (HE) staining was used to observe the pathological morphology of renal tissue, Masson staining for renal fibrosis, and TUNEL staining for renal cell apoptosis.

### Target protein IRE1α-DMDD small molecule docking

For the docking of the target protein, IRE1α-DMDD, with small molecule: firstly, the target protein information was obtained through the UniProt database, and the three-dimensional structure of the protein was modeled in PyMOL using the amino acid sequence; Protein crystal structures for key targets were obtained from the PDB database (PDB:5HGI, https://doi.org/10.2210/pdb5hgi/pdb). Subsequently, software was used to predict the possible active sites on the protein and to identify the ligand binding site. Finally, the target protein and the small molecule ligand files were prepared using the docking software, and the target protein IRE1α was docked with the DMDD small molecule to obtain a preliminary docking phase structure. The phase with the optimal docking energy was selected for structure extraction in the docking process. The highest docking energy was scored, and the docking structure diagram was visualized using PyMOL software.

### RNA-seq analysis

RNA samples were prepared to establish a target gene sample library to control the relevant data quality. By comparing it with the reference genome, the sample relationship was analyzed. Enrichment analyses on differential genes, Gene Ontology (GO) analysis, and Kyoto Encyclopedia of Genes and Genomes (KEGG) pathways analysis were conducted to identify the significant differences in target genes and elucidate the signaling pathway enriched by the target protein.

### Cell proliferation

The cytotoxicity of DMDD was determined using the Cell Counting Kit-8 (CCK8) (211104Z01-10, Shanghai Bai Sai Biotechnology Co., Ltd, China). HK-2 cells were seeded in a 96-well plate at 8 × 103 cells/well density. After 24 h of administration and before measuring the optical density (OD) with a microplate reader (Shimadzu, UV-1900), 10 μL of CCK-8 reagent was added to each well, and the cells were incubated for 40 min.

### Flow cytometry detection

Apoptosis was detected using the Annexin V-FITC and PI kits (No. GY03J22P4305, Wuhan Elite Biotechnology Co., Ltd., China). HK-2 cells were seeded in a six-well plate, digested with EDTA-free trypsin, centrifuged, and then the supernatant was discarded. Diluted 1 × Annexin V Binding Buffer was added to the resulting solution and stained with FITC and PI for 20 min. On-machine testing was performed using flow cytometry (Accuri C6 Plus, BD Company, USA), and data analysis was conducted using FlowJo software.

### Transmission electron microscopy (TEM)

Tissue and HK-2 cells were immobilized in 3% glutaraldehyde at 4 ℃ overnight, followed by three 10-min washes with 0.1 mol/L phosphate buffer. Samples were then post-fixed in 1% osmium acid for 2 h. The dehydrating agent was removed using fractionated alcohol and acetone dehydration, and the sections were then embedded and double-stained with uranyl acetate. Finally, the ER and autophagic structures were observed using a transmission electron microscope (TEM) (Hitachi, Ltd., Tokyo).

### Confocal microscope observation

HK-2 cells were cultured in a laser confocal dish at a density of 3 × 10^4^ cells/well, followed by transfection with mRFP-GFP-LC3 adenovirus according to the instructions above. The adenovirus was administered to the various groups for 24 h, and the color changes of mRFP and GFP were observed using laser confocal microscopy (fv300, Olympus, Japan).

### Immunofluorescence

HK-2 cells were cultured in 24-well plates, fixed with 4% paraformaldehyde for 15 min after administration, and infiltrated with 0.5% Triton X-100 for 10 min. After blocking with 10% goat serum for 1 h, the cells were incubated overnight at 4 °C with primary antibodies of α-SMA (No. AC220613007, Wuhan Sevier Biotechnology Co., Ltd., China), Vimentin (No. 22133349, Wuhan Sevier Biotechnology Co., Ltd., China), and E-cadherin (No. AC220613009, Proteintech Group, USA). The cells were further incubated with DyLight 593/614 (Proteintech, China) for 1 h at room temperature. Finally, HK-2 cells were visualized using a laser confocal microscopy after adding a drop of DAPI-containing mounting agent to the cell climbing sheet. The results are presented in Fig. 5A–C.

### ELISA

For the in vivo experiments, the levels of IL-1β and IL-10 in kidney tissues were detected according to the kit instructions of enzyme-linked Immunosorbent assay (ELISA) (Jingmei Biotech, China). In the in vitro experiments, the supernatant from HK-2 cells was collected to measure the levels of TGF-β1, hyaluronic acid, hematin, collagen type III, and collagen type IV. All experiments were performed in triplicate. The results are depicted in Fig. 5D–M.

### Western blotting analysis

The total protein of tissues and cells was extracted using PMSF-containing RIPA lysate. Each group's protein concentration was determined using a bicinchoninic acid assay (BCA), and the proteins were separated using SDS-PAGE electrophoresis before being transferred to the Polyvinylidene Fluorid (PVDF) membranes. Subsequently, membranes coated with different primary antibodies were incubated overnight at 4 ℃. The primary antibodies used included: β-actin, BAX, Bcl-2, GRP78, CHOP, Beclin1, ATG5, LC3II/I, JNK, IRE1α (1:1000, Proteintech, China); p-mTOR, mTOR, p-CaMKKβ, CaMKKβ, p-AMPK, AMPK, p-JNK (1:1000, Cell Signaling Technology, USA); and p-IRE1α (1:1000, DMDDam, UK). Further incubation was performed with a 1:10000 dilution of secondary antibodies (Invitrogen, USA) for 30–60 min. Finally, protein bands were visualized using the Odyssey infrared fluorescence imaging system (Licor, USA) and ImageJ software.

### Transfection of overexpression lentivirus

HK-2 cells were subjected to lentiviral infection (Genechem, China). After determining the multiplicity of infection (MOI) value and screening the concentration of puromycin (21313814, Guangzhou Shuopu Biotechnology Co., Ltd., China) in the pre-experiment, the transfected HK-2 cells were inoculated into a 60 mm cell culture dish in the formal experiment. When the cell density was 70–80%, HK-2 cells were treated according to the experimental group. The cells were divided into three experimental groups: the normal group, the high glucose group, and the DMDD high dose group (8 μmol/L). The normal group was cultured in low-glucose DMEM complete medium, with the final concentration of puromycin at 2 μg/mL. The high glucose group and the administration group were modeled using high-glucose DMEM complete medium with the final concentration of puromycin at 2 μg/mL. Three days after the infection, intracellular fluorescence expression was observed under an inverted microscope. After administration, cells were collected for validation using western blotting.

### Statistical analysis

The experimental data were analyzed with SPSS 26.0 (IBM Corporation, USA), expressed as mean ± standard deviation. One-way ANOVA was used for multigroup comparisons, and LSD was used for pairwise comparisons between groups. *P* < 0.05 was considered statistically significant.

## Results

### DMDD-mediated improvement in renal function injury of C57BL/6 J mice

In order to verify the renal protective effect of DMDD, we treated different groups of C57BL/6 J mice with different concentrations of DMDD for 8 weeks after establishing an in vivo DN model. Body weight is an important indicator of diabetic patients. Patients with High blood glucose, due to insufficient insulin secretion or insulin resistance, cannot fully absorb and utilize glucose, but resorts to decomposing fat and protein for energy, resulting in excessive protein consumption and weight loss. It was found in the study that the weight of mice in the DN group decreased compared to that of the control group. Notably, mice in the nifedipine + DMDD group exhibited significantly higher body weights than those in the DN group (Fig. 1C). Prior to DMDD administration, the blood glucose levels of mice in each treatment group were higher than that in the control group, indicating that the diabetic model was successfully established in this experiment. After DMDD administration, the blood glucose levels of mice in each treatment group were significantly reduced (Fig. 1D). In addition, the kidney index (KI = kidney weight (mg) / body weight (g), normal kidney index > 45%) was significantly higher in the DN group compared to the control group. KI was significantly decreased in different DMDD administration groups (Fig. 1E). In further in vivo experiments, we also measured renal function indicators in mice, Urea Nitrogen (UN), PRO, and Scr (Fig. 1F, G, H). Compared to the control group, the DN group exhibited increased levels of PRO, Scr, and BUN, suggesting impaired renal function. After DMDD treatment, the above indicators were significantly down-regulated. The above results suggest that DMDD has a protective effect against renal function damage in DN mice and may slow the progression of DN (Fig. 1F, G, H). Compared to the control group, the contents of TC, TG and LDL in the DN group were increased, and the content of HDL was decreased. Similarly, compared to the DN group, the contents of TC, TG and LDL in the DMDD group were decreased, and the content of HDL was increased (Fig. 1I, J, K, L). It is suggested that DMDD can effectively mitigate renal injury in DN mice.

### DMDD-induced amelioration of renal histopathological damage in C57BL/6 J mice

**Fig. 2:**
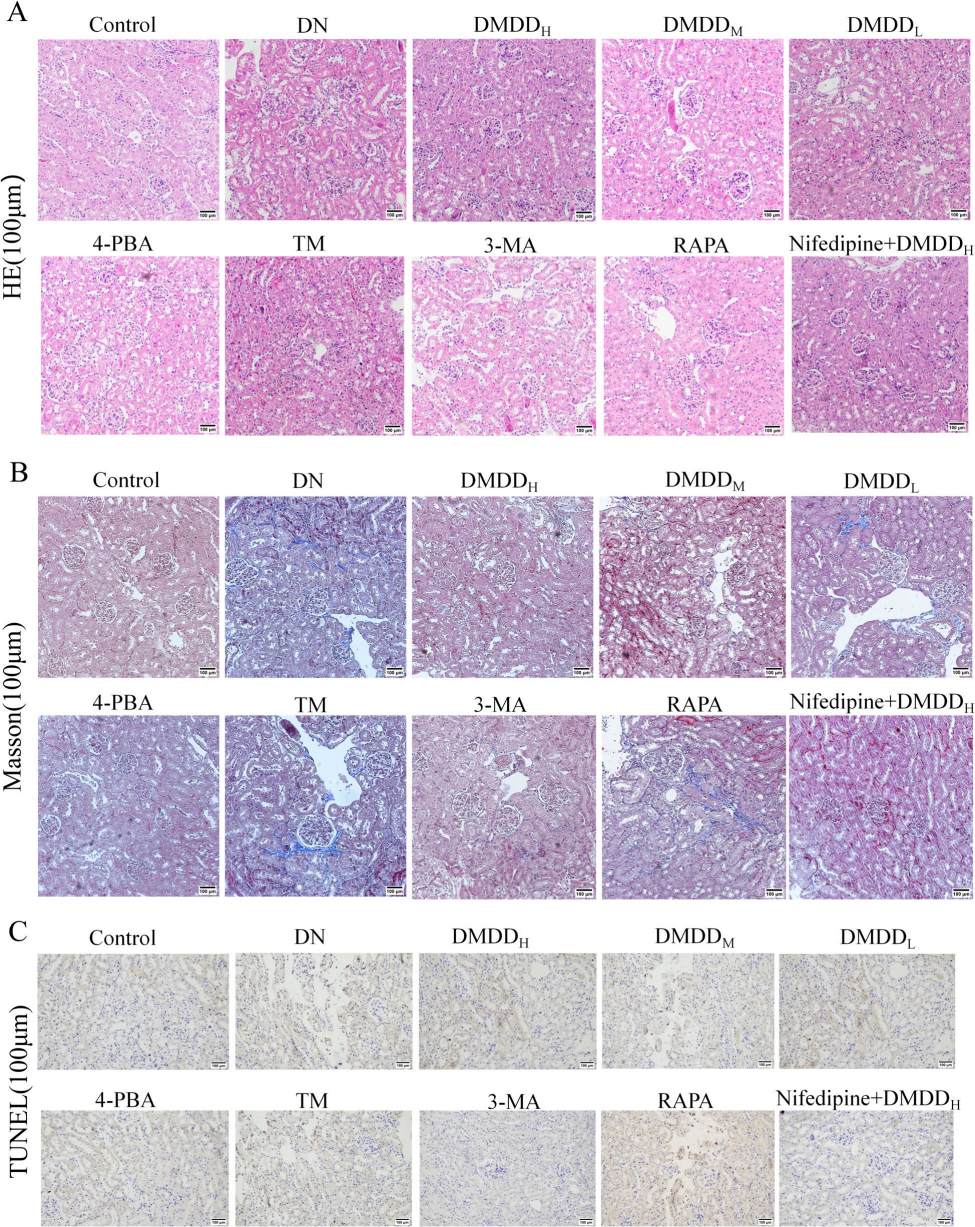
Effect of DMDD on histopathological kidney damage in DN mice. **A** HE staining. showing reduced cell infiltration. Scale bars 100 μm. **B** Masson staining. Scale bars 100 μm. **C** TUNEL staining. Scale bar " -" = 100 μm. The Control group denotes the normal group, DN group denotes the diabetic nephropathy model group, DMDD_L_, DMDD_M_, and DMDD_H_ represent low-, medium-, and high-dose DMDD groups, respectively, 4-PBA group indicates endoplasmic reticulum stress inhibitor 4- PBA group, TM group denotes the endoplasmic reticulum stress agonist TM group, 3-MA group denotes the autophagy inhibitor 3-MA group, RAPA group denotes the autophagy agonist RAPA group, and Nifedipine + DMDD_H_ group denotes the nifedipine group + DMDD high-dose group. (n = 8)

Histological HE staining observation revealed that the renal tubules of normal control mice were tightly arranged with clear borders. Compared to control mice, DN mice showed mild vacuolization of renal tubular epithelial cells, disorganized glomerular structure, unclear borders, obvious thickening, and inflammatory cell infiltration in the renal interstitium. The vacuolization of renal tubules, disorganized glomerular structure, and inflammatory cell infiltration were reduced in different dose groups of DMDD, 4-PBA, 3-MA, and nifedipine + DMDD_H_, and cell infiltration was reduced (Fig. 2A). Masson staining showed that compared to the control group, glomeruli and intertubules from DN mice exhibited obvious blue collagen fiber deposition. After the intervention of different dose groups of DMDD, 4-PBA, 3-MA, and nifedipine + DMDD_H_, there was a significant decrease in the number of blue-stained collagen fibers in the renal tubules and glomeruli, and the degree of fibrosis was reduced significantly (Fig. 2B). TUNEL staining demonstrated that renal tissue apoptosis was significantly increased in the DN group compared to the control group and decreased in the different dose groups of DMDD, the 4-PBA group, the 3-MA group, and the nifedipine + DMDD_H_ group after the treatment, suggesting that DMDD could reduce apoptosis in the kidneys of the mice and attenuate the pathological damage of their kidneys (Fig. 2C).

### Insights of mechanism into DMDD's therapeutic effects on DN revealed by IRE1α-DMDD docking and transcriptome analysis

**Fig. 3:**
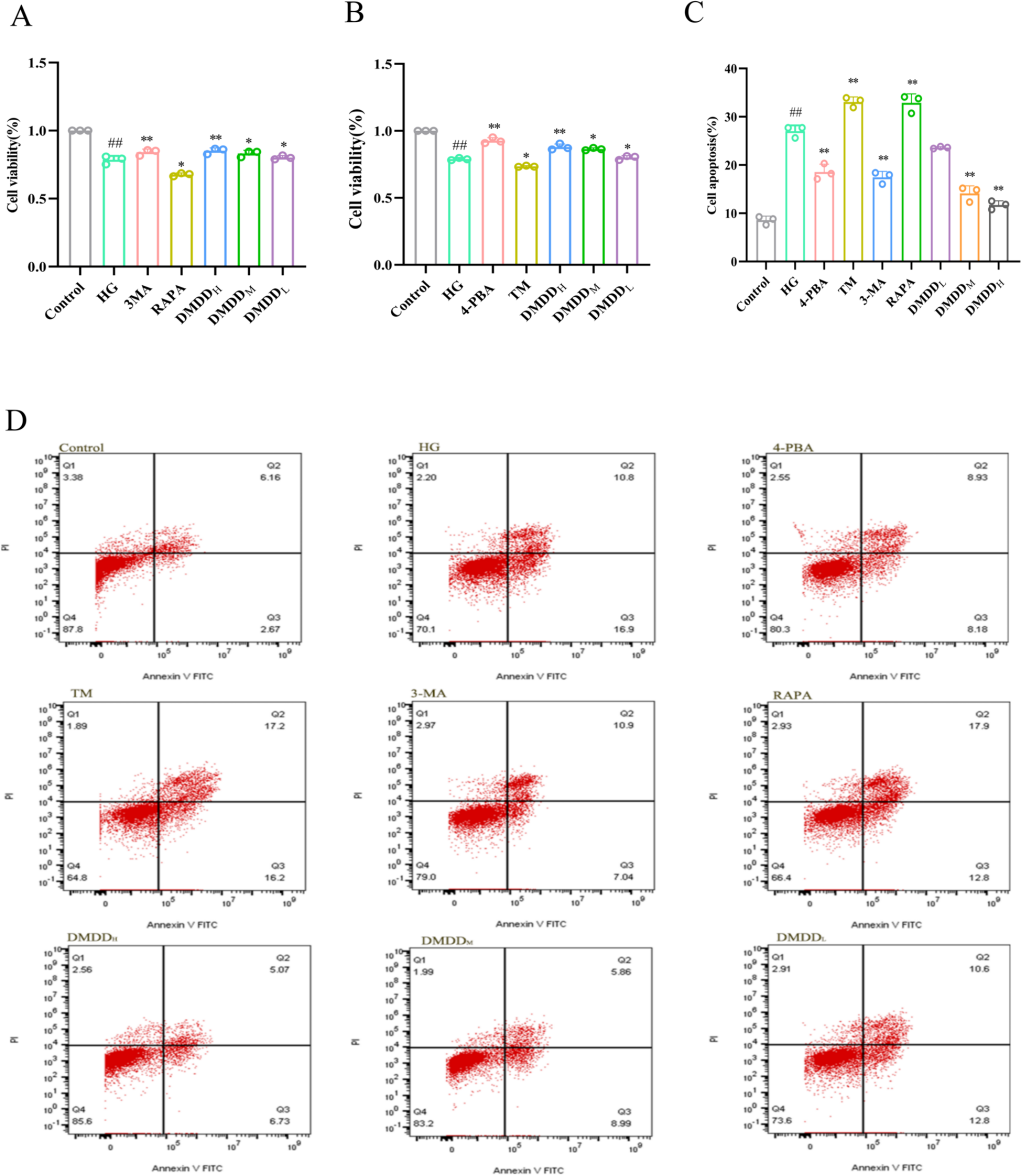
Docking results and transcriptional analysis of the target protein IRE1α and DMDD. **A** 3D structure of IRE1α constructed using PyMol-based amino acid sequence; **B** Visualization of the 7 active centers of IRE1α predicted by software; **C** Visualization of the 7 docking pockets of IRE1α with specific conformations; **D** Green fluttering bands in the figure represent the IRE1α target proteins, with the yellow stick indicating the DMDD small molecule structure. **E** After the docking between DMDD and IRE1α was completed, relevant calculations were conducted to determine the docking results data. **F** Docking scores for IRE1α activity pockets are shown in the figure; **G** Wayne plots. **B** indicates the normal group, **C** indicates the model group, and M indicates the model group; **H** GO enrichment analysis. **I** KEGG enrichment bar graph

In order to further verify the target and specific mechanism of DMDD in the treatment of DN, we carried out the docking of the target protein IRE1α with DMDD. We conducted a transcriptome analysis based on the ERS-autophagy signaling pathway. In this study, IRE1α, an important protein of ERS, was selected to dock with DMDD. The docking results revealed that IRE1α docked with DMDD, forming seven potential active sites, and the active center was visualized (Fig. 3A–D). Finally, the structure corresponding to the phase with the optimal docking energy was subsequently extracted. The highest docking score between IRE1α and the small molecules at the TARGET_BOX 3 binding site was -5.50 kcal/mol; with Pocket 3 identified as the optimal binding pocket, together indicating that DMDD could bind to IRE1α, thereby exerting the therapeutic effect by regulating the IRE1α-related signaling pathway (Fig. 3E, F). According to the results from animal experiments, we selected the high-dose DMDD treatment group with the best therapeutic effect for the transcriptome study. Using a *p-*value threshold of 0.05 and an absolute value of log2FC greater than 1.5, the up-regulated and down-regulated genes of the blank group, the model group, and the administration group were screened. As shown in Fig. 3G, compared to the model group, there were 1366 significantly up-regulated genes and 571 significantly down-regulated genes in the administration group, indicating that DMDD can significantly change the expression of kidney genes in DN mice. The GO enrichment analysis of the administration group and the model group is depicted in Fig. 3I. Regarding biological processes, the differentially expressed genes were mainly enriched in pathways related to the regulation of cell cycle, and cellular components were primarily enriched in chromosomes, membranes, and organelles. In terms of molecular function, the most substantial enrichment was observed for genes implicated in ATP binding. KEGG enrichment analysis was performed on the differentially expressed genes. Both the significantly up-regulated and down-regulated genes in the administration group and the model group were enriched in autophagy-related signaling pathways. These pathways were ranked according to their *p* values, from most to least significant. As shown in Fig. 3H, the genes with significant changes in expression were mainly enriched in the cell cycle, autophagy signaling pathway, and metabolic pathway.

### DMDD-induced alleviation of inflammatory response in DN mice

Long-term hyperglycemic environment associated with DN is prone to inflammatory responses [13]. ELISA results indicated that the secretion levels of IL-10 were lower in the DN group compared to the control group, while the secretion levels of IL-1β were increased. Treatment with DMDD restored IL-10 levels and inhibited the secretion of IL-1β. This indicates that DMDD effectively mitigates the inflammatory response caused by DN (Fig. 1M, N).

### DMDD-enhanced viability of HK-2 cells

**Fig. 4:**
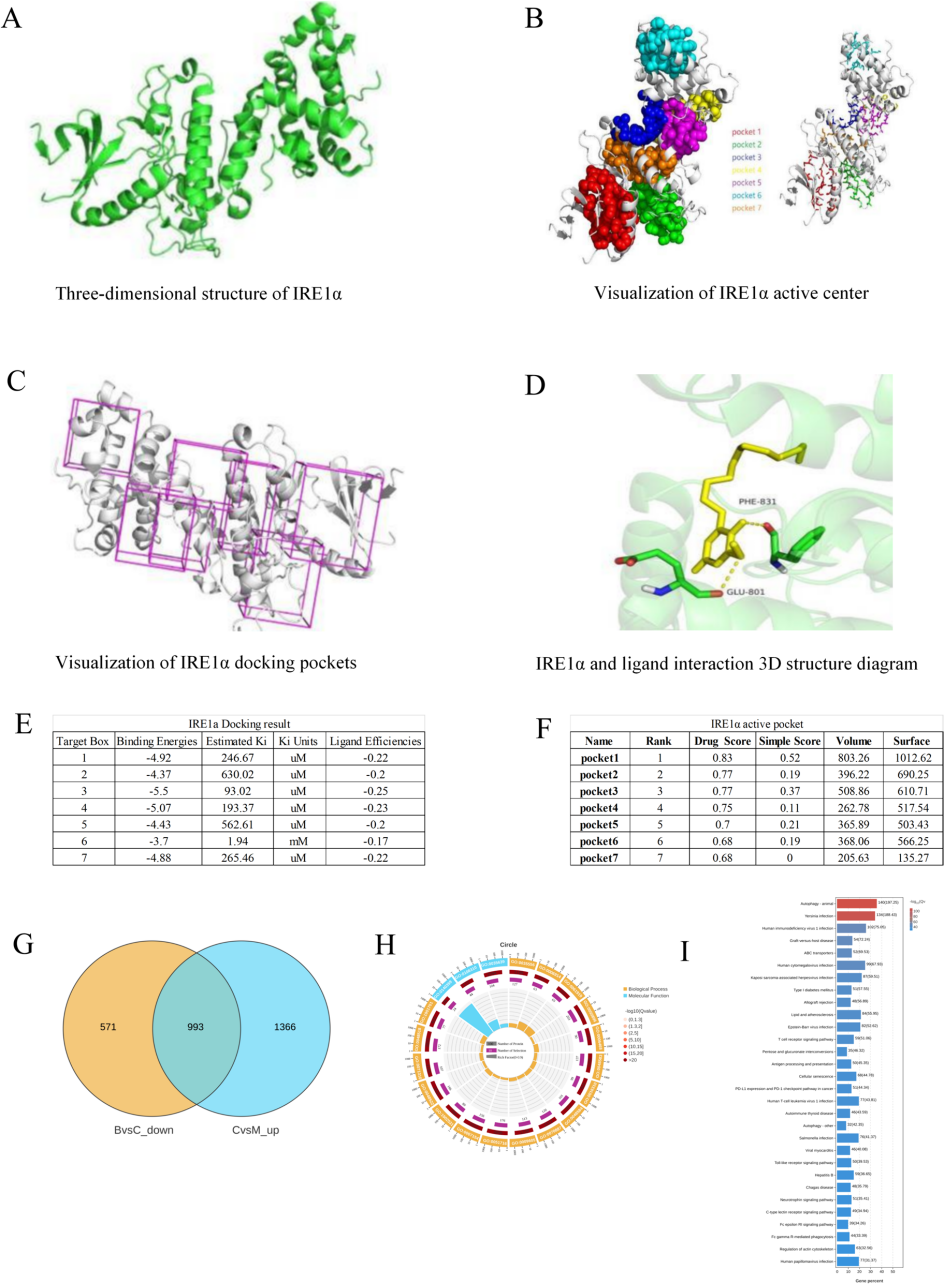
Effects of DMDD on HK-2 cells. **A** Effects of 3-MA group, RAPA group and different DMDD dosages HK-2 cell viability; **B** Effects of different doses of 4-PBA group, TM group and DMDD administration group on HK-2 cell viability; **C** Effect of DMDD on the apoptotic rate of HK-2 cells; **D** Flow cytometry analysis of DMDD’s effect on the apoptosis of HK-2 cells. The Control group denotes the normal group, the HG group denotes the HK-2 high-glucose model group, DMDD_L_, DMDD_M_, and DMDD_H_ represent low-, medium-, and high-dose DMDD groups, respectively, 3-MA group represents autophagy inhibitor 3-MA group, and RAPA group denotes the autophagy agonist RAPA group. Data are expressed as mean ± SD (n = 8). Statistical significance: compared to the normal group, ^#^*P* < 0.05, ^##^*P* < 0.01; compared to the model group, *P < 0.05, **P < 0.01

The proliferation activity of HK-2 cells was detected by the Cell Counting Kit-8 (CCK8) method. The results demonstrated that high glucose (HG) conditions inhibited HK-2 cell proliferation compared to the control group. However, the inhibitory effect of HG on cell proliferation was significantly weakened after administration of 3-MA, RAPA, 4-PBA, TM, and different doses of DMDD (Fig. 4A, B).

### DMDD-inhibited the apoptosis of HK-2 cells

Flow cytometry analysis revealed that the apoptosis rate of HK-2 cells in the HG group was higher than that in the control group. DMDD treatment markedly reduced the apoptosis rate compared to the HG group, demonstrating its potential cytoprotective effects. TM and RAPA were used as ERS and autophagy agonists, respectively. The apoptosis rate was augmented in both groups, indicating that excessive ERS and autophagy may induce apoptosis (Fig. 4C, D).

### DMDD-inhibited high glucose-induced fibrosis in HK-2 cells

**Fig. 5:**
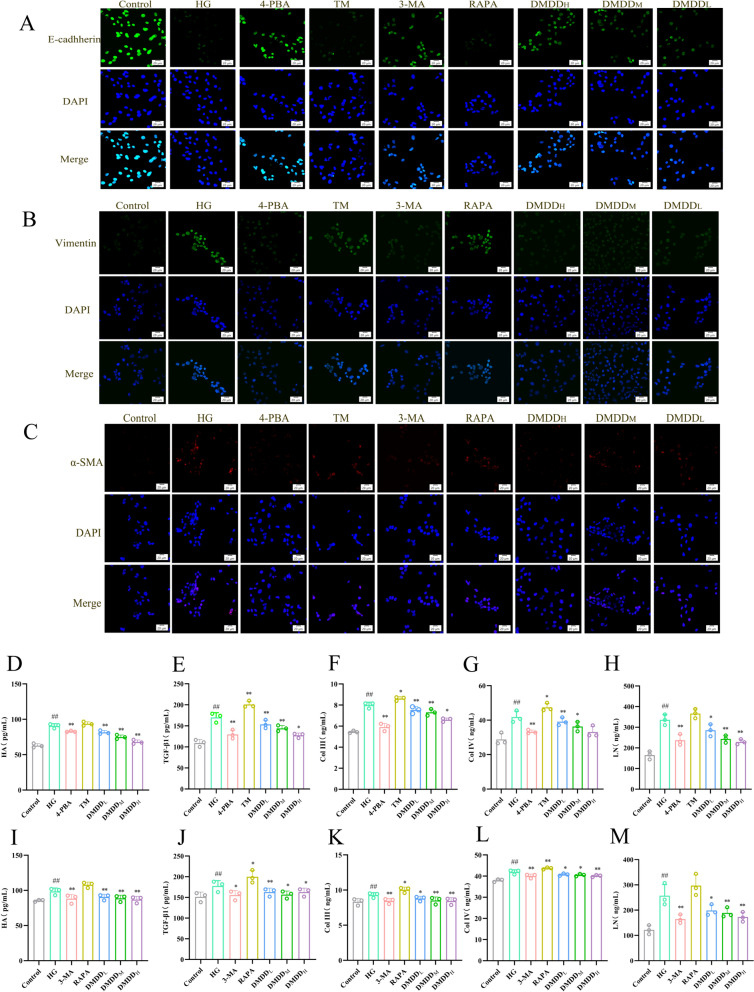
Effect of DMDD on epithelial-mesenchymal transition in HK-2 cells. **A**–**C** Immunofluorescence detection results; **D**–**M** Effect of DMDD on cellular pro-fibrotic factors. The Control group indicates the normal group, HG group indicates the high-glucose group, DMDD_L_, DMDD_M_, and DMDD_H_ represent low-, medium-, and high-dose DMDD groups, respectively, 4-PBA group indicates the low-dose group of DMDD, and 4-PBA group indicates the high-dose group of DMDD. group, 4-PBA group denotes the endoplasmic reticulum stress inhibitor 4-PBA group, TM group denotes the endoplasmic reticulum stress agonist TM group, 3-MA group denotes the autophagy inhibitor 3-MA group, and RAPA group denotes the autophagy agonist RAPA group. Data are expressed as mean ± SD (n = 8). Statistical significance: compared to the normal group, ^#^*P* < 0.05, ^##^*P* < 0.01; compared to the model group, *P < 0.05, **P < 0.01

During in vitro experiments, the expression levels of the E-cadherin, Vimentin, and α-SMA markers were detected using immunofluorescence. As shown, HG inhibited E-cadherin expression, while DMDD upregulated the expression. In contrast, HG increased the α-SMA and Vimentin levels, while DMDD inhibited the overexpression of α-SMA and Vimentin (Fig. 5A–C). In addition, ELISA data determined that the secretion levels of TGF-β1, HA, LN, Col III, and Col IV in the HG group were higher than those in the control group, and the levels of the above indexes were dramatically reduced after DMDD treatment (Fig. 5D–M).

### DMDD-induced significant reduction of ERS degree

**Fig. 6:**
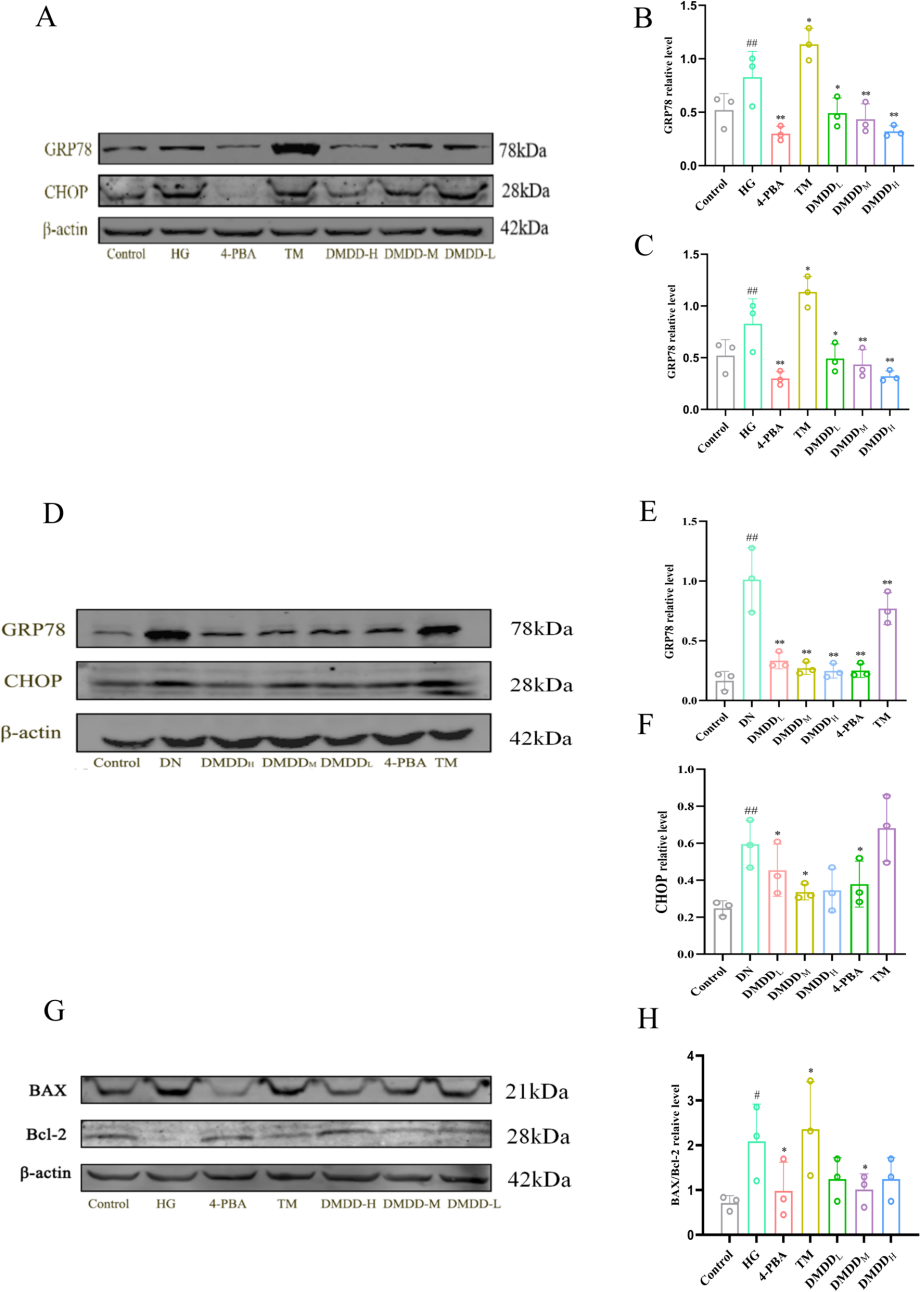
Effect of DMDD on the expression levels of GRP78, CHOP, BAX, Bcl-2 proteins. **A**–**C** Effect of DMDD on the expression levels of GRP78 and CHOP proteins in kidney tissues of DN mice. **D**–**F** Effects of DMDD on the expression levels of GRP78 and CHOP proteins in high glucose-induced HK-2 cells. **G**, **H** Effect of DMDD on the expression levels of BAX and Bcl-2 proteins in high-glucose-induced HK-2 cells. The Control group indicates the normal group, HG group indicates the high glucose group, DMDD_L_ group indicates the DMDD low-dose group, DMDD_M_ group indicates the DMDD medium-dose group, DMDD_H_ group indicates the DMDD high-dose group, 4-PBA group indicates the endoplasmic reticulum stress inhibitor 4-PBA group, and TM group indicates the endoplasmic reticulum stress agonist TM group. Data are demonstrated as mean ± SD (n = 8), statistical significance: compared to the normal group, ^#^P < 0.05, ^##^*P* < 0.01; compared to the model group, *P < 0.05, **P < 0.01

**Fig. 7:**
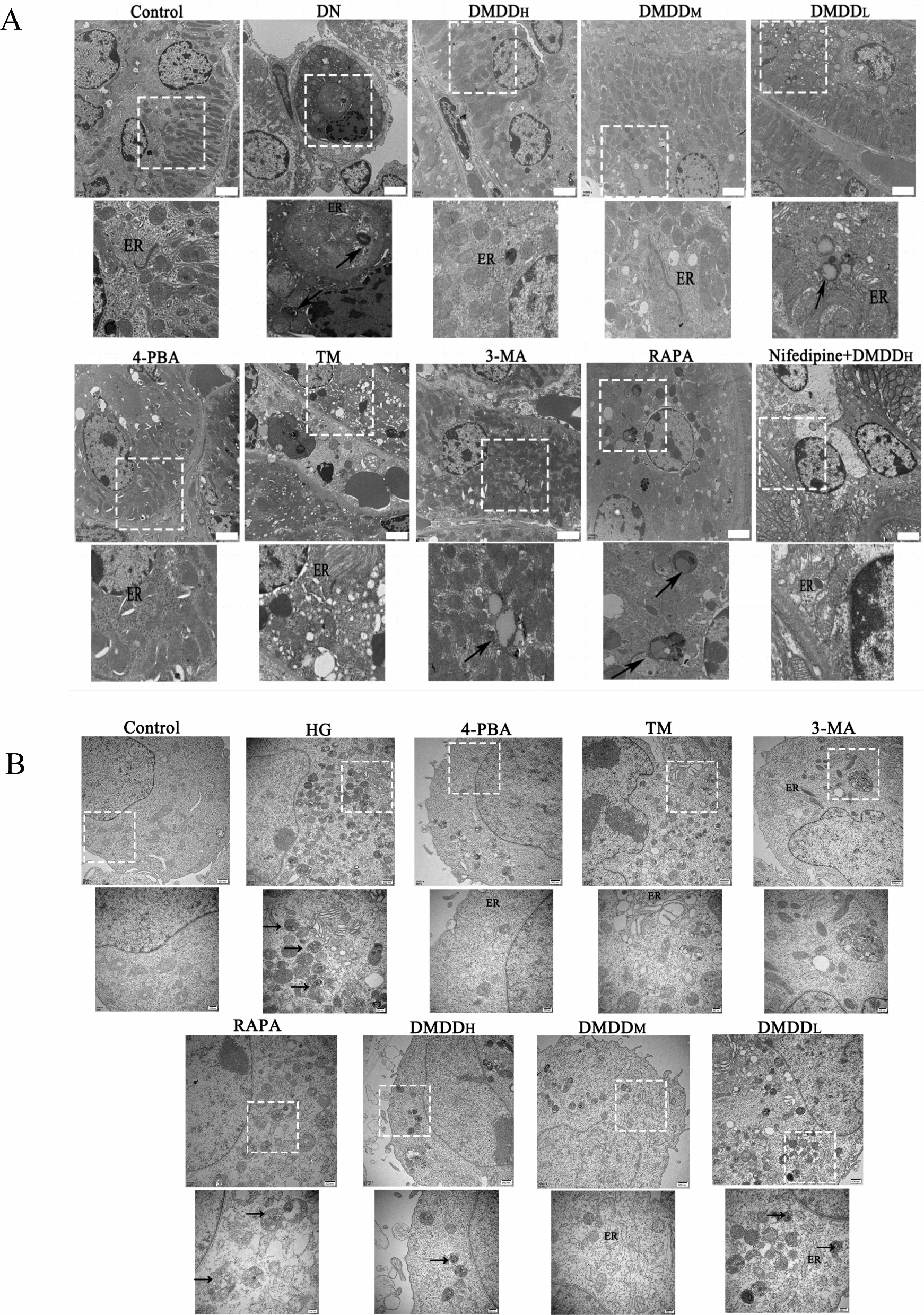
Effects of DMDD on the ultrastructure of kidneys and HK-2 cells in DN mice. **A** Effects of DMDD on the ultrastructure of kidney in DN mice; **B** Effects of DMDD on the ultrastructure of HK-2 cells. "Black arrow" represents autophagic vesicles or autophagic lysosomes; "ER" denotes endoplasmic reticulum. The control group denotes the normal group, DN group denotes the diabetic nephropathy mouse model group, HG group denotes the high glucose HK-2 cells group, DMDD_L_ group denotes the DMDD low-dose group, DMDD_M_ group denotes DMDD medium-dose group, DMDD_H_ group denotes DMDD high-dose group, 4-PBA group denotes 4-PBA group, TM group denotes the endoplasmic reticulum stress inhibitor group, TM group denotes the endoplasmic reticulum stress agonist group, 3-MA group denotes the autophagy inhibitor group, 3-MA group, RAPA group denotes the autophagy agonist (RAPA) group, and Nifedipine + DMDD_H_ group denotes nifedipine + DMDD high-dose group

**Fig. 8:**
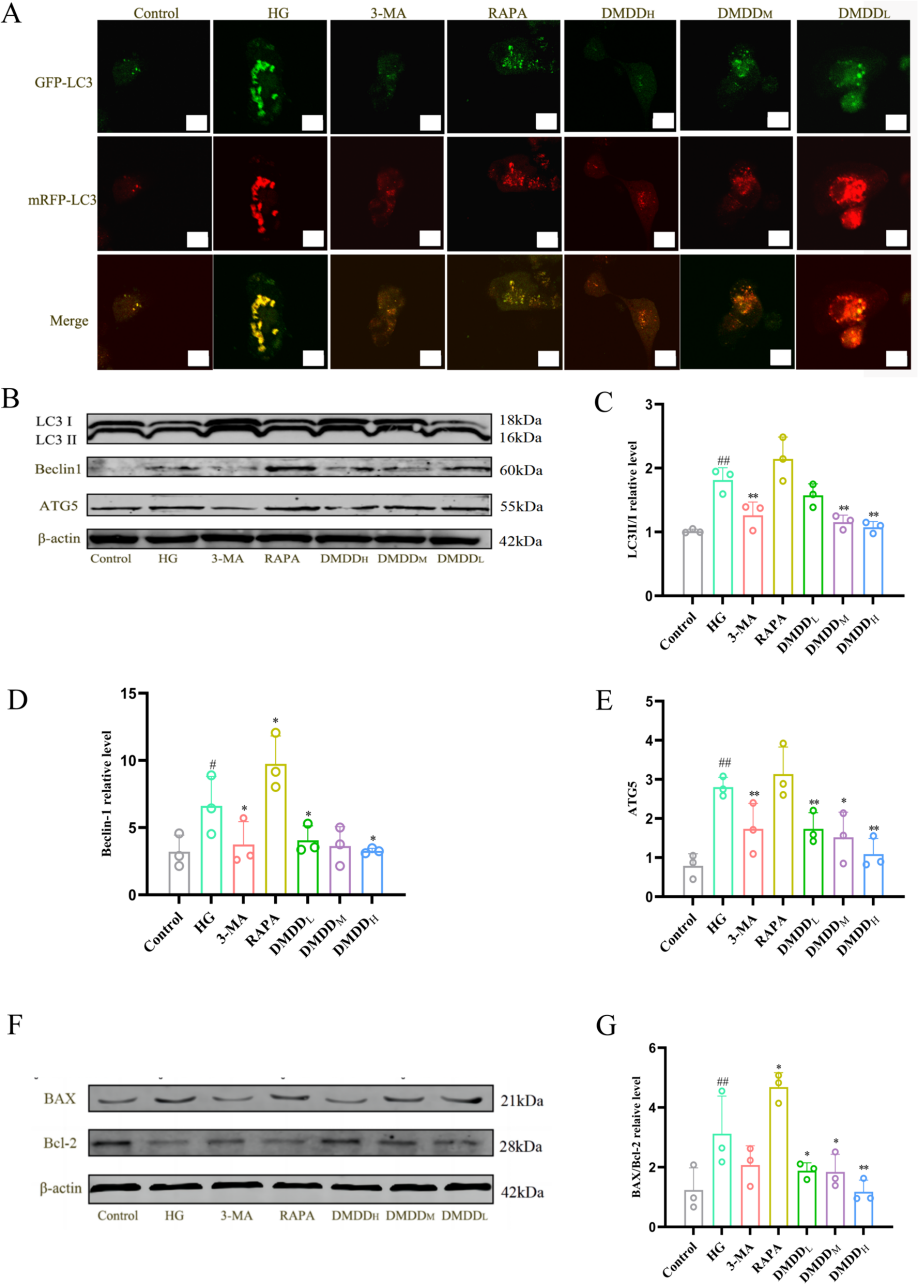
Effect of DMDD on HK-2 cells. **A** Observation of autophagic flux by confocal laser scanning microscopy. GFP fluoresces shows green, and mRFP fluoresces red under a laser confocal microscope; **B**–**E** Effects of DMDD on the expression levels of autophagy-related proteins LC3I, LC3II, Beclin 1, and ATG 5 in high glucose-induced HK-2 cells. The Control group signifies the normal group, HG group treatment the HK-2 high-glucose model group, DMDD_L_ indicates the DMDD low-dose group, DMDD_M_ indicates the DMDD medium-dose group, DMDD_H_ represents the DMDD high-dose group, 3-MA indicates the autophagy inhibitor 3-MA group, and RAPA indicates the autophagy agonist RAPA group. Data are demonstrated as mean ± SD (n = 8), statistical significance: compared to the normal group, ^#^*P* < 0.05, ^##^*P* < 0.01; compared to the model group, *P < 0.05, **P < 0.01

Western blotting analysis demonstrated that the expression levels of GRP78 and CHOP in the model group was significantly higher than that in the control group. DMDD could reverse the abnormal expression of GRP78 and CHOP in the 4-PBA group, showing effects comparable to those of the ERS inhibitor (Fig. 6A–F). Both in vivo and in vitro experiments revealed that high glucose conditions induce severe ERS, and DMDD significantly decreases the degree of ERS. TEM results showed that the ER in the model group was swollen and vacuolated, with significant attenuation of the ER swelling observed following DMDD treatment (Fig. 7A, B). The BAX/Bcl-2 ratio in the HG group was significantly increased, suggesting that HG-induced ERS may lead to apoptosis (Fig. 8F, G).

### DMDD-inhibited autophagy and reduced apoptosis

To explore the impact of DMDD on autophagy, both in vivo and in vitro experiments were conducted. TEM observations revealed an increased number of autophagosomes and autolysosomes in the model group compared to the control group. However, the number of autophagosomes and autolysosomes in the DMDD and 3-MA treatment groups was significantly reduced. In addition, cells were transfected with mRFP-GFP-LC3 adenovirus to visualize autolysosome formation. As shown in Fig. 8A, HG conditions enhanced the formation of red dots. They promoted the maturation of autolysosomes in HK-2 cells, whereas DMDD treatment reduced these red autophagic lysosomal spots similar to the effect observed with 3-MA treatment. Moreover, in the experiment, the expression levels of LC3II/I, Beclin1, and ATG5 in the model group increased, and decreased after DMDD intervention (Fig. 8B–E). Western blotting results showed that BAX/Bcl-2 ratio increased in the model group and decreased after treatment with 3-MA and DMDD, suggesting that DMDD inhibits autophagy and reduces apoptosis (Fig. 8F–G).

### DMDD-induced reduction of autophagy through decreased Ca^2+^ concentration and IRE1α pathway inhibition

To verify the mechanism of ERS-induced autophagy, the Ca^2+^ imbalance pathway was first studied in vivo. Western Blot showed that, compared to the control group, the expression levels of p-mTOR, p-AMPK, and p-CaMKKβ were significantly increased in the DN group. On the contrary, these abnormalities were significantly reversed in the DMDD treatment group and the combined treatment group receiving nifedipine and DMDD (Nifedipine + DMDD_H_) (Fig. 5G–K). Additionally, TEM results showed that autophagosomes and autolysosomes were significantly reduced in the DMDD and the nifedipine + DMDD_H_ treatment groups, indicating a synergistic effect of DMDD and nifedipine in reducing autophagy (Fig. 7B).

**Fig. 9:**
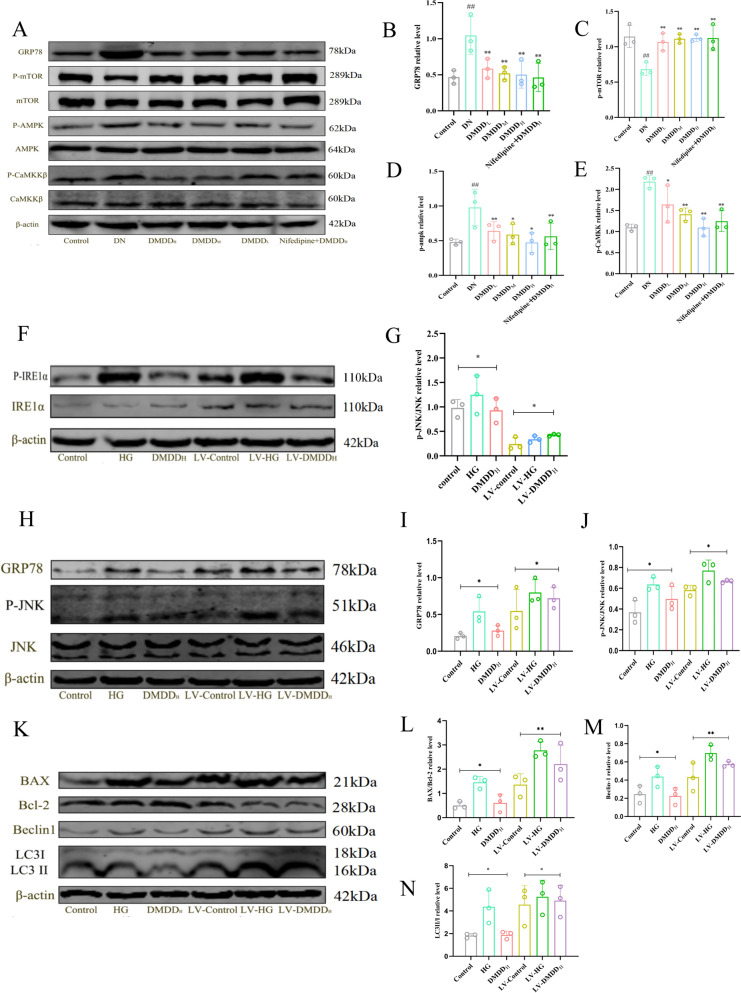
Effect of DMDD on the expression of ERS-autophagy signaling pathway protein levels in mouse kidney tissues. **A**–**E** Effect of different dosage administration groups of DMDD on the expression levels of GRP78, p-mTOR, mTOR, p-AMPK, AMPK, p-CaMKKβ, CaMKKβ proteins in kidney tissues of DN mice. **F**–**G** Effects of different dosage administration groups of DMDD on the expression levels of p-IRE1α and IRE1α proteins in high glucose-induced HK-2 cells after transfection. **H**–**J** Effect of varying dosage administration groups on the protein expression levels of GRP78, p-JNK, and JNK in high glucose-induced HK-2 cells after transfection. expression levels. **K**–**N** Effect of different dosage groups of DMDD on the protein expression levels of Beclin-2, LC3 I, and LC3 II in high glucose-induced HK-2 cells after transfection. Note: The Control group indicates the normal group, the HG group indicates the high-glucose model group of HK-2 cells, the LV-DMDD_L_ group indicates the low-dose transfected DMDD group DMDD, and the LV -DMDD_M_ group denotes the DMDD medium-dose transfected DMDD group, and LV-DMDD_H_ group denotes the high-dose transfected DMDD group after transfection. Data are performed as mean ± SD (n = 8), statistical significance: compared to the normal group, ^#^*P* < 0.05, ^##^*P* < 0.01; compared to the model group, *P < 0.05, **P < 0.01

In addition, the IRE1α pathway induced by ERS is related to autophagy, so we detected the expression levels of IRE1α pathway-related proteins in HK-2 cells infected with IRE1α lentivirus in vitro. As shown in Fig. 9F, G, after the transfection of IRE1α by HK-2 cells in the control group, the expression level of IRE1α and p-IRE1α increased. Compared to the untransfected cell group, the ratio of p-IRE1α/IRE1α was decreased in the DMDD group, indicating that DMDD treatment inhibits the expression of IRE1α, thereby suppressing apoptosis and autophagy. Furthermore, overexpression of IRE1α led to increased levels of p-JNK, Beclin1, and LC3II/I compared to untransfected cells. However, following high glucose (HG) treatment, no significant changes in the levels of p-IRE1α, p-JNK, Beclin1, and LC3II/I were observed between the HG and DMDD treatment groups (Fig. 9H–N).

These findings collectively demonstrate that the combination of nifedipine and DMDD can inhibit persistent ERS through the CaMKKβ/AMPK/MTOR signaling pathway, regulate intracellular calcium homeostasis, and subsequently reduce autophagy, thereby exerting cytoprotective effects.

## Discussion

DN accounts for about 40% of end-stage renal disease, and may progress to chronic renal failure, and even lead to death in diabetic patients [14, 15]. However, the complex pathogenesis of DN continues to pose significant treatment challenges. Averrhoa carambola root, a characteristic Chinese herbal medicine native to Guangxi, exhibits a range of pharmacological activities, including hypoglycemic, hypolipidemic, anti-inflammatory, anticancer and other effects. DMDD, a compound extracted by our research group has demonstrated potential therapeutic benefits in diabetes [16], DN [11], breast cancer [17], lung cancer [18], Alzheimer 's disease [18] and many other diseases. Recent studies have shown that ERS is readily induced under conditions of high glucose or elevated free fatty acids [19, 20]. High glucose levels can trigger inflammatory responses in renal tubular epithelial cells, leading to extracellular matrix deposition and cell-mesenchymal transition, which aggravates renal tubular interstitial fibrosis [21, 22]. Studies have shown that there is some interaction between ERS and autophagy. Cells' intracellular and extracellular stimulation triggers ERS, activating the UPR to remove misfolded or unfolded proteins in the ER. However, when these stimuli persist, the URP cannot remove misfolded or unfolded proteins in time. In such cases, ERS initiates autophagy, relieving the cellular pressure caused by the accumulation of ER protein, thus maintaining intracellular homeostasis. However, the persistent presence of ERS will increase the level of autophagy, resulting in the excessive decomposition of intracellular substances, ultimately causing cell damage and even cell death [23, 24]. Study have shown that ERS-triggered autophagy is affected by the regulation of Ca^2+^ storage and release by the ER [25]. Therefore, we speculated that the activation of ERS and autophagy may be crucial factors of DN [26]. In order to verify this inference, a DN mouse model was established using a high-fat and high-sugar diet combined with intraperitoneal injection of STZ. The results revealed that the levels of PRO, Scr and BUN in the model group were significantly increased, suggesting that HG induces renal damage. Notably, DMDD treatment resulted in substantial improvement of these renal impairments. Kintoko K et al. [16] reported that DMDD significantly reduced FBG levels in DN mice, which is consistent with our experimental results. In addition, Shunyu Lu et al. [11] similarly found that DMDD not only reduced blood glucose levels and reversed the abnormal expression levels of Scr and BUN in their study on DMDD’s ameliorative effects on DN through the TLR4/MyD88/NF-κB pathway [11]. Similarly, our study demonstrated that DMDD significantly reduced Scr and BUN levels, thereby attenuating renal injury in DN mice. This evidence proved that HG plays an important role in renal tubular injury, HG-induced atrophy and apoptosis of HK-2 cells is an important factor in renal failure, and DMDD can significantly ameliorate this abnormality [27]. HG plays an important role in renal tubular injury, with HG-induced atrophy and apoptosis of HK-2 cells being critical contributors to renal failure [27]. Therefore, HG-induced HK-2 injury was employed as an in vitro model of DN.

The typical characteristics of DN include the accumulation of extracellular matrix proteins [28] and pathological tubulointerstitial fibrosis [29]. Excessive deposition of collagen fibers is an important feature of renal fibrosis. The results of Masson staining showed increased collagen fibers in the DN group compared to the DMDD treatment group, where collagen deposition was significantly reduced, suggesting the presence of renal fibrosis in diabetic mice. Study have found that type IV collagen is highly expressed in DN mice [30]. The secretion level of pro-fibrotic factors in vitro was also detected by ELISA. The data showed that the levels of TGF-β1, hyaluronic acid, laminin, type III collagen and type IV collagen in the DMDD treatment group were significantly lower than those in the HG group. These findings suggest that DMDD effectively reduces collagen accumulation and mitigates fibrosis in HK-2 cell. EMT of renal tubular cells is a key step in the occurrence of renal interstitial fibrosis [31]. In this study, immunofluorescence was used to visualize the EMT of HK-2 cells. The results showed that E-cadherin was down-regulated, Vimentin and α-SMA were up-regulated in HG group. Similarly, long-term exposure to high glucose also induces inflammation, leading to renal hypertrophy and persistent damage of renal tubules [32]. Shunyu Lu et al. [11], in studying the improvement of DN by DMDD through the TLR4/MyD88/NF-κB pathway, found that DN was associated with inflammatory response, and the levels of IL-6 and TNF-α in DN mice were suppressed after intervention with DMDD, which proved that DMDD could effectively improve the inflammatory response in DN mice. Similarly, in vitro ELISA results also indicated that DMDD inhibited IL-1β secretion and increased IL-10 secretion, that is, DMDD could improve the inflammatory response in DN mice. In vitro ELISA results showed that DMDD inhibited the IL-1β secretion and enhanced IL-10 secretion. The pathological changes in the model group were consistent with the DN performance, confirming that the DN model has been established, and DMDD can improve high glucose-induced renal fibrosis.

ERS is closely related to DN kidney damage, as previous studies have observed ERS activation in the kidneys of DN patients [33]. The misfolded proteins caused by HG and excessive nutrient intake in patients can lead to misfolded protein accumulation, further aggravating ERS and potentially triggering apoptosis [34]. The up-regulation of GRP78 expression is considered to be a marker of ERS activation [35]. Typically, moderate ERS stimulation allows cells to maintain homeostasis. However, excessive ERS due to hyperglycemia results in GRP78 binding to defective proteins, activating the UPR, and increasing CHOP expression, which can lead to cell damage and even death [36–38]. Our results show that DMDD treatment, both in vitro and in vivo, significantly down-regulated the expression levels of GRP78 and CHOP, indicating that DMDD protects the kidney and renal tubular cells by inhibiting ERS.

Autophagy, an important mechanism for cellular stability, undergoes various changes throughout its development. The autophagosomes observed through TEM and the autolysosomal changes detected via laser confocal microscopy are considered gold standards for confirming autophagy. Under normal circumstances, HK-2 cells exhibit low autophagy activity; however, high glucose conditions can activate autophagy in DN patients due to nutrient changes [39]. In order to explore the potential mechanism by which DMDD inhibits autophagy, we studied autophagy-related proteins both in vivo and in vitro. The results show that DMDD reduced the levels of LC3II / I, Beclin1 and ATG5, thereby decreasing the number of autophagosomes and autolysosomes. These data suggest that the protective effect of DMDD on DN is attributed to the reduction of autophagy.

It is worth mentioning that both ERS and autophagy play crucial roles in the occurrence and progression of DN, and exploring the interaction between the two may offer new therapeutic directions for DN treatment. ERS can directly regulate autophagy through the IRE1α pathway, or indirectly through intracellular Ca^2+^ concentration IRE1α, a key component of UPR, upregulates the expression of multiple UPR-related genes. GRP78 is dissociated from the transmembrane protein IRE1α, resulting in the phosphorylation of IRE1α to p-IRE1α in vivo. And the activated p-IRE1α triggers the IRE1α pathway involved in autophagy regulation, which can transcriptionally activate Beclin1 and innitiate autophagy.

What is more, the phosphorylation of JNK, a downstream target of IRE1α, can attenuate the inhibitory effect of Bcl-2 on Beclin1, promoting both autophagy and apoptosis [40]. HK-2 cells transfected with LV-IRE1α in vitro exhibited significant up-regulation of p-JNK, Beclin1 and LC3II / I under high glucose conditions. However, there was no significant change in the levels of p-JNK, Beclin1 and LC3II / I protein in HK-2 cells transfected with LV-IRE1α after treated with DMDD. Calcium homeostasis plays a key role in maintaining protective function, and ERS-induced release of calcium ions from the ER into the cytoplasm can induce autophagy. Increased concentration of Ca^2+^ activates the calcineurin CaMKKβ, which subsequently phosphorylates the downstream AMPK protein and inhibits the expression of mTOR, leading to the conversion of LC3I to LC3II [40, 41], thus enhancing autophagy. Moreover, there is a correlation between Ca^2+^ concentration and Beclin1, with Beclin1 further up-regulating autophagy. This study found that the expression of p-CaMKKβ, p-AMPK, Beclin1 and LC3II / I in DN group was up-regulated, while the p-mTOR levels were down-regulated. Conversely, DMDD treatment significantly decreased the levels of p-CaMKKβ, p-AMPK, Beclin1, LC3II / I and increased the p-mTOR levels in the kidneys of mice, suggesting that DMDD effectively inhibits autophagy.

## Conclusion

**Fig. 10:**
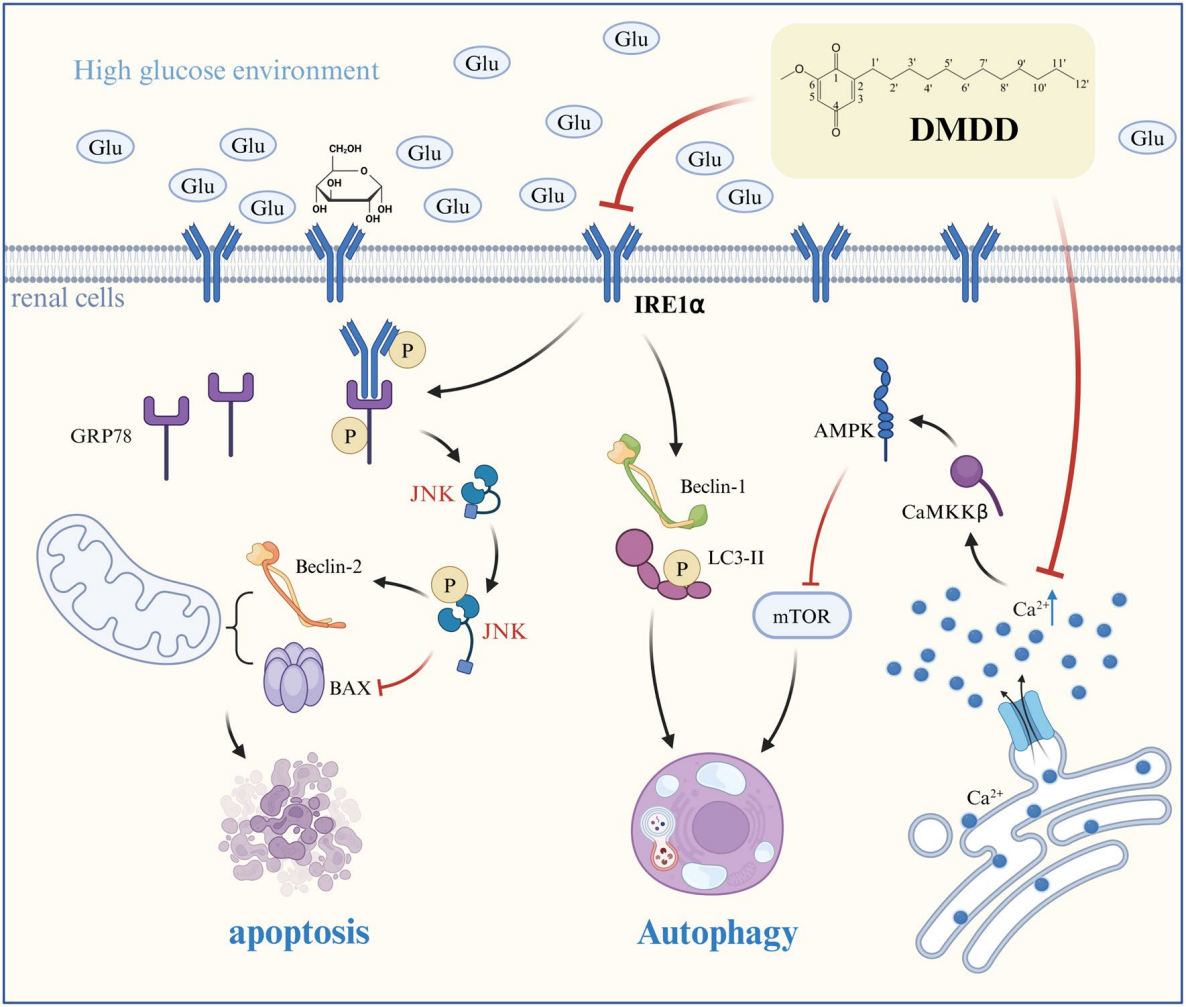
DMDD alleviates the mechanism of ERS-autophagy in HK-2 cells induced by high glucose and diabetic nephropathy in mice. The figure shows that DMDD mitigates excessive ER stress and autophagy by reducing Ca^2+^ concentration and inhibiting CaMKKβ and IRE1α pathways. Consequently, this intervention alleviates kidney damage, including proteinuria and renal fibrosis, thereby delaying the onset and progression of DN

In summary, HG induces severe ERS, which in turn leads to extensive autophagy and subsequent apoptosis. Our study demonstrates that DMDD significantly inhibits HG-induced ERS and autophagy, further reducing apoptosis. Therefore, we propose that DMDD may exert its protective effects on renal tubular epithelial cells and mitigates renal injury by inhibiting the IRE1α pathway and the CaMKKβ/AMPK/MTOR pathway, thereby reducing ERS-induced autophagy (see Fig. 10 for the specific mechanism diagram).

## Data Availability

All data generated from this study are included in this article. Further enquiries can be directed to the corresponding author.

## Abbreviations

DMDD: 2-Dodecyl-6-methoxycyclohexa-2, 5-diene-1, 4-dione
STZ: Streptozotocin
HG: Hyperglycemia
DN: Diabetic nephropathy
ER: Endoplasmic reticulum
ERS: Endoplasmic reticulum stress
UPR: Unfolded protein response
EMT: Epithelial-mesenchymal transition
4-PBA: 4-Phenylbutyric acid
TM: Tunicamycin
MA: 3-Methyladenine
RAPA: Rapamycin
FBG: Fasting blood glucose
BUN: The blood urea nitrogen
Scr: Serum creatinine
PRO: Proteinuria
TG: Triglyceride
CHO: Total cholesterol
HDL-C: High-density lipoprotein cholesterol
LDL-C: Low-density lipoprotein cholesterol
HE: Hematoxylin–eosin

## References

[1] Haraguchi R, Kohara Y, Matsubayashi K, Kitazawa R, Kitazawa S. New insights into the pathogenesis of diabetic nephropathy: proximal renal tubules are primary target of oxidative stress in diabetic kidney. Acta Histochem Cytochem. 2020;53(2):21–31. 32410750 10.1267/ahc.20008 PMC7212204

[2] Liu BC, Tang TT, Lv LL, Lan HY. Renal tubule injury: a driving force toward chronic kidney disease. Kidney Int. 2018;93(3):568–79. 29361307 10.1016/j.kint.2017.09.033

[3] Eriguchi M, Lin M, Yamashita M, et al. Renal tubular ACE-mediated tubular injury is the major contributor to microalbuminuria in early diabetic nephropathy. Am J Physiol Renal Physiol. 2018;314(4):F531-f542. 29187372 10.1152/ajprenal.00523.2017 PMC5966765

[4] Yuan D, Liu XM, Fang Z, et al. Protective effect of resveratrol on kidney in rats with diabetic nephropathy and its effect on endoplasmic reticulum stress. Eur Rev Med Pharmacol Sci. 2018;22(5):1485–93. 29565511 10.26355/eurrev_201803_14497

[5] Liu Y, Xu D, Wang L, et al. MBTPS2 exacerbates albuminuria in streptozotocin-induced type I diabetic nephropathy by promoting endoplasmic reticulum stress-mediated renal damage. Arch Physiol Biochem. 2022;128(4):1050–7. 32255378 10.1080/13813455.2020.1749084

[6] Kume S, Yamahara K, Yasuda M, et al. Autophagy: emerging therapeutic target for diabetic nephropathy. Semin Nephrol. 2014;34(1):9–16. 24485025 10.1016/j.semnephrol.2013.11.003

[7] Sankrityayan H, Oza MJ, Kulkarni YA, et al. ER stress response mediates diabetic microvascular complications. Drug Discovery Today. 2019;24(12):2247–57. 31430543 10.1016/j.drudis.2019.08.003

[8] Koch EAT, Nakhoul R, Nakhoul F, et al. Autophagy in diabetic nephropathy: a review. Int Urol Nephrol. 2020;52(9):1705–12. 32661628 10.1007/s11255-020-02545-4

[9] Chen F, Sun Z, Zhu X, et al. Astilbin inhibits high glucose-induced autophagy and apoptosis through the PI3K/Akt pathway in human proximal tubular epithelial cells. Biomed Pharmacother. 2018;106:1175–81. 30119185 10.1016/j.biopha.2018.07.072

[10] Ni Z, Lin X, Wen Q, et al. WITHDRAWN: Effect of 2-dodecyl-6-methoxycyclohexa-2, 5-diene-1, 4-dione, isolated from Averrhoa carambola L. (Oxalidaceae) roots, on advanced glycation end-product-mediated renal injury in type 2 diabetic KKAy mice. Toxicol Lett. 2021;339:88–96. 33423876 10.1016/j.toxlet.2020.11.022

[11] Lu S, Zhang H, Wei X, et al. 2-dodecyl-6-methoxycyclohexa-2,5-diene-1,4-dione isolated from Averrhoa carambola L. root ameliorates diabetic nephropathy by inhibiting the TLR4/MyD88/NF-κB pathway. Diabetes Metab Syndr Obes. 2019 Aug 7; 12: 1355–1363. 10.2147/DMSO.S209436. Erratum in: Diabetes Metab Syndr Obes. 2020 Dec 16; 13: 5015. 10.2147/DMSO.S209436 PMC6689538 31496773

[12] Li J, Pang Q, Huang X, et al. 2-Dodecyl-6-Methoxycyclohexa-2, 5-Diene-1, 4-Dione isolated from Averrhoa carambola L. root inhibits high glucose-induced EMT in HK-2 cells through targeting the regulation of miR-21–5p/Smad7 signaling pathway. Biomed Pharmacother. 2024;172:116280. 10.1016/j.biopha.2024.116280. 38368837 10.1016/j.biopha.2024.116280

[13] Zhang H, Wei X, Lu S, et al. Retraction notice to “Protective effect of DMDD, isolated from the root of Averrhoa carambola L., on high glucose induced EMT in HK-2 cells by inhibiting the TLR4-BAMBI-Smad2/3 signaling pathway” [Biomed. Pharmacother. 113 (2019) 108705]. Biomed Pharmacother. 2024;171:116196. 38296729 10.1016/j.biopha.2024.116196

[14] Chen SJ, Lv LL, Liu BC, et al. Crosstalk between tubular epithelial cells and glomerular endothelial cells in diabetic kidney disease. Cell Prolif. 2020;53(3):e12763. 31925859 10.1111/cpr.12763 PMC7106959

[15] Keri KC, Samji NS, Blumenthal S. Diabetic nephropathy: newer therapeutic perspectives. J Community Hosp Intern Med Perspect. 2018;8(4):200–7. 30181826 10.1080/20009666.2018.1500423 PMC6116149

[16] Kintoko K, Xu X, Lin X, Huang R, et al. Hypoglycaemic activity of 2-dodecyl-6-methoxycyclohexa-2,5-diene-1,4-dione in streptozotocin-induced diabetic mice through ameliorating metabolic function and regulating peroxisome proliferator-activated receptor γ. Arch Med Sci. 2018;14(5):1163–72. 30154901 10.5114/aoms.2016.63285 PMC6111351

[17] Li H, Zhu X, Zhang J, Shi J. MicroRNA-25 inhibits high glucose-induced apoptosis in renal tubular epithelial cells via PTEN/AKT pathway. Biomed Pharmacother. 2017;96:471–9. 29031207 10.1016/j.biopha.2017.10.019

[18] Wang L, Yang X, Song Q, et al. Uncovering the pharmacological mechanism of 2-Dodecyl-6-methoxycyclohexa-2,5 -Diene-1,4-Dione against lung cancer based on network pharmacology and experimental evaluation. Front Pharmacol. 2021;12:617555. 33613291 10.3389/fphar.2021.617555 PMC7887632

[19] Wei X, Xu X, Chen Z, et al. Protective effects of 2-Dodecyl-6-Methoxycyclohexa-2,5 -Diene-1,4-Dione isolated from Averrhoa Carambola L. (Oxalidaceae) roots on neuron apoptosis and memory deficits in Alzheimer’s disease. Cell Physiol Biochem. 2018;49(3):1064–73. 30196278 10.1159/000493289

[20] Sage AT, Holtby-Ottenhof S, Shi Y, et al. Metabolic syndrome and acute hyperglycemia are associated with endoplasmic reticulum stress in human mononuclear cells. Obesity (Silver Spring). 2012;20(4):748–55. 21633399 10.1038/oby.2011.144

[21] Lhotak S, Sood S, Brimble E, et al. ER stress contributes to renal proximal tubule injury by increasing SREBP-2-mediated lipid accumulation and apoptotic cell death. Am J Physiol Renal Physiol. 2012;303(2):F266–78. 22573382 10.1152/ajprenal.00482.2011

[22] Fu B, Yang J, Chen J, et al. Preventive effect of Shenkang injection against high glucose-induced senescence of renal tubular cells. Front Med. 2019;13(2):267–76. 29700792 10.1007/s11684-017-0586-8

[23] Han J, Pang X, Shi X, et al. Ginkgo biloba extract EGB761 ameliorates the extracellular matrix accumulation and mesenchymal transformation of renal tubules in diabetic kidney disease by inhibiting endoplasmic reticulum stress. Biomed Res Int. 2021;2021:6657206. 33860049 10.1155/2021/6657206 PMC8009711

[24] Senft D, Ronai ZA. UPR, autophagy, and mitochondria crosstalk underlies the ER stress response. Trends Biochem Sci. 2015;40(3):141–8. 25656104 10.1016/j.tibs.2015.01.002 PMC4340752

[25] Qi Z, Chen L. Endoplasmic reticulum stress and autophagy. Adv Exp Med Biol. 2019;1206:167–77. 31776985 10.1007/978-981-15-0602-4_8

[26] Feng N, Wang B, Cai P, et al. ZEA-induced autophagy in TM4 cells was mediated by the release of Ca(2+) activates CaMKKbeta-AMPK signaling pathway in the endoplasmic reticulum. Toxicol Lett. 2020;323:1–9. 31982503 10.1016/j.toxlet.2020.01.010

[27] Loeffler I, Wolf G. Epithelial-to-mesenchymal transition in diabetic nephropathy: fact or fiction? Cells. 2015;4(4):631–52. 26473930 10.3390/cells4040631 PMC4695850

[28] Gewin LS. Renal fibrosis: primacy of the proximal tubule. Matrix Biol. 2018;68–69:248–62. 29425694 10.1016/j.matbio.2018.02.006 PMC6015527

[29] Zou C, Liu X, Liu R, et al. Effect of the oral iron chelator deferiprone in diabetic nephropathy rats. J Diabetes. 2017;9(4):332–40. 27121697 10.1111/1753-0407.12420

[30] Li J, Liu H, Takagi S, et al. Renal protective effects of empagliflozin via inhibition of EMT and aberrant glycolysis in proximal tubules. JCI Insight. 2020;5(6):e129034. 32134397 10.1172/jci.insight.129034 PMC7213787

[31] Liao L, Song M, Li X, et al. E3 ubiquitin ligase UBR5 Drives the growth and metastasis of triple-negative breast cancer. Can Res. 2017;77(8):2090–101. 10.1158/0008-5472.CAN-16-2409 28330927

[32] Liu Z, Han Y, Zhao F, et al. Nobiletin suppresses high-glucose-induced inflammation and ECM accumulation in human mesangial cells through STAT3/NF-κB pathway. J Cell Biochem. 2019;120(3):3467–73. 30499124 10.1002/jcb.27621

[33] Yao F, Li Z, Ehara T, et al. Fatty acid-binding protein 4 mediates apoptosis via endoplasmic reticulum stress in mesangial cells of diabetic nephropathy. Mol Cell Endocrinol. 2015;411:232–42. 25958041 10.1016/j.mce.2015.05.003

[34] Belali OM, Ahmed MM, Mohany M, et al. LCZ696 protects against diabetic cardiomyopathy-induced myocardial inflammation, ER stress, and apoptosis through inhibiting AGEs/NF-κB and PERK/CHOP signaling pathways. Int J Mol Sci. 2022;23(3):1288. 35163209 10.3390/ijms23031288 PMC8836005

[35] Ju Y, Su Y, Chen Q, et al. Protective effects of Astragaloside IV on endoplasmic reticulum stress-induced renal tubular epithelial cells apoptosis in type 2 diabetic nephropathy rats. Biomed Pharmacother. 2019;109:84–92. 30396095 10.1016/j.biopha.2018.10.041

[36] Shao DC, Xue H, Lu LM. The role of endoplasmic reticulum stress in diabetic nephropathy. Sheng li ke xue jin zhan [Progress in physiology]. 2015;46(2):111–5. 26201109

[37] Hetz C, Papa FR. The unfolded protein response and cell fate control. Mol Cell. 2018;69(2):169–81. 29107536 10.1016/j.molcel.2017.06.017

[38] Koya D, Kitada M, Kume S, et al. Interventions against nutrient-sensing pathways represent an emerging new therapeutic approach for diabetic nephropathy. Clin Exp Nephrol. 2014;18(2):210–3. 24221306 10.1007/s10157-013-0908-3

[39] Granato M, Romeo MA, Tiano MS, et al. Bortezomib promotes KHSV and EBV lytic cycle by activating JNK and autophagy. Sci Rep. 2017;7(1):13052. 29026157 10.1038/s41598-017-13533-7 PMC5638868

[40] Hoxhaj G, Hughes-Hallett J, Timson RC, et al. The mTORC1 signaling network senses changes in cellular purine nucleotide levels. Cell Rep. 2017;21(5):1331–46. 29091770 10.1016/j.celrep.2017.10.029 PMC5689476

[41] Fedeli C, Filadi R, Rossi A, et al. PSEN2 (presenilin 2) mutants linked to familial Alzheimer disease impair autophagy by altering Ca(2+) homeostasis. Autophagy. 2019;15(12):2044–62. 30892128 10.1080/15548627.2019.1596489 PMC6844518

